# Willingness to pay for HIV pre- and post-exposure prophylaxis services delivered via an online pharmacy in Kenya

**DOI:** 10.1186/s12913-025-12766-x

**Published:** 2025-04-22

**Authors:** Michalina A. Montaño, Yilin Chen, Enrique M. Saldarriaga, Nicholas Thuo, Catherine Kiptinness, Andy Stergachis, Melissa L. Mugambi, Kenneth Ngure, Katrina F. Ortblad, Monisha Sharma

**Affiliations:** 1 Vaccine and Infectious Diseases Division (VIDD), Fred Hutchinson Cancer Center, Seattle, USA; 2 The Comparative Health Outcomes, Policy, and Economics (CHOICE) Institute, University of Washington, Seattle, USA; 3 Partners in Health and Research Development, Centre for Clinical Research, Kenya Medical Research Institute, Nairobi, Thika, Kenya; 4 Department of Global Health, University of Washington, Seattle, USA; 5 School of Public Health, Jomo Kenyatta University of Agriculture and Technology, Juja, Kenya; 6 Public Health Science Division, Fred Hutchinson Cancer Center, Seattle, USA

**Keywords:** PrEP, PEP, HIV prevention, Willingness to pay, Pharmacies, Online delivery

## Abstract

**Background:**

HIV pre- and post-exposure prophylaxis (PrEP/PEP) provision via online pharmacies could expand reach of HIV prevention in Eastern and Southern Africa, but designing sustainable delivery models will require assessing the amount potential users are willing to pay for online PrEP/PEP provision.

**Methods:**

We administered willingness to pay (WTP) questionnaires to both potential online PrEP users and current online PrEP/PEP users in Nairobi, Kenya using a stated preference approach to measure the amount participants were willing to pay for PrEP/PEP service delivery components. Participants ≥ 18 years were recruited via banner ads on an online pharmacy website on pages displaying sexual health products. We used multivariable gamma regression models to assess characteristics associated with differences in mean WTP for a 30-day PrEP or 28-day PEP course (including HIV self-testing, remote clinical consultation, drugs, and delivery fees).

**Results:**

From May 2022 and December 2023, 1,512 participants completed WTP questionnaires: 772 potential online PrEP users and 740 current online PrEP/PEP users. Most participants (98.3%, 1486/1,512) were willing to pay some amount for online PrEP services. For a one-month PrEP supply, potential online PrEP users were willing to pay 1388 KSH ($11.77 USD) and current online PrEP/PEP users were willing to pay 1271.2 KSH ($10.77 USD) on average. Most current online PrEP/PEP users (81.4%, 602/740) were also willing to pay for online PEP services; for a 28-day PEP supply, they were willing to pay 812.9 KSH ($6.89 USD) on average. Among potential online PrEP users, male sex, current enrollment in school, high income, and a history of online pharmacy purchases were associated with higher WTP for PrEP. Among current online PrEP/PEP users, higher income and prior online pharmacy purchases were associated with higher WTP for PrEP, and older age (> 24) and prior online pharmacy purchases were associated with higher WTP for PEP.

**Conclusion:**

Most potential and current online PrEP/PEP users in Nairobi were willing to pay for online pharmacy-based PrEP/PEP and demonstrated similar WTP. Providing PrEP/PEP through online pharmacies may sustainably expand coverage of these HIV prevention services.

**Supplementary Information:**

The online version contains supplementary material available at 10.1186/s12913-025-12766-x.

## Introduction

Despite remarkable advancements in HIV treatment and prevention, an estimated 500,000 new HIV infections occurred in 2022 in Eastern and Southern Africa [1]. Oral pre-exposure prophylaxis (PrEP) is a highly effective HIV prevention strategy recommended by the World Health Organization (WHO) since 2015 and has been available in government clinics free of charge in Kenya since 2017 [2–5]. However, uptake of clinic-based PrEP in Kenya and other regions of Eastern and Southern Africa (ESA) is low [6]; PrEP users report access barriers including long wait times, lack of privacy, stigma, and inconvenient clinic hours [7, 8]. WHO guidelines for differentiated and simplified PrEP implementation emphasize the need for person- and community-centered PrEP delivery approaches to increase PrEP accessibility. Previous studies have demonstrated that providing PrEP in community settings such as retail pharmacies can achieve high uptake [9, 10]. Additionally, qualitative research and reviews found that stakeholders considered PrEP service delivery through retail pharmacies in ESA to be acceptable and feasible [11, 12]. Similarly, post-exposure prophylaxis (PEP) is a promising HIV prevention tool when started within 72 h after a potential exposure [13]. Few studies have assessed the impact of making PEP available outside of clinics, although modeling suggests that community-based PEP services can be efficient for HIV prevention [14].

In addition to brick-and-mortar settings in the community, leveraging online pharmacies for PrEP/PEP service delivery is a novel strategy that could expand the reach of HIV biomedical prevention. Online pharmacies offer convenient and discreet delivery of medications, health products, and remote clinical consultations directly to consumers. Telehealth services in Kenya and other countries of Africa are rapidly expanding due to growing internet and cell phone use; expansion was expedited by the COVID- 19 pandemic.—which revealed the advantages of online service provision over in-person encounters [15, 16]. A proposed care pathway for online PrEP/PEP delivery starts with clients ordering an HIV test which is delivered to their preferred location. Online PrEP/PEP could be supported with HIV self-testing (HIVST), which was recently recommend by the WHO to support PrEP initiation and continuation [17]. Next, clients complete a telehealth consultation with remote clinician to ensure PrEP/PEP is not contraindicated. After verification of HIV-negative status, clients, receive a PrEP/PEP prescription with drugs delivered location of their choice. Client also have access to ongoing virtual support in case of questions/concerns [18]. Our team recently conducted a pilot study of online PrEP/PEP service delivery in Kenya utilizing this pathway and found it achieved high uptake among eligible clients and was acceptable among clients and providers [19].

However, online pharmacy PrEP/PEP provision will require sustainable models for service delivery as online pharmacies are private retailers which sell products and services to promote client health. To inform the scale-up and sustainability of PrEP/PEP service delivery via online retail pharmacies, we aim to understand how much potential clients are willing to pay for components of the model (e.g., HIVST) to inform price points that are accessible to clients and profitable to pharmacies. To our knowledge no prior studies have assessed willingness to pay (WTP) for online PEP or PrEP services in Africa.

## Methods

### Study population and setting

We used data from two separate studies of online PrEP and PEP delivery in Nairobi County, Kenya. From May to November 2022, we conducted a cross-sectional survey and discrete choice experiment (DCE) to understand preferences for online PrEP delivery among online pharmacy clients. Then, from November 2022 to December 2023, we conducted a pilot of daily oral PrEP and PEP delivered via an online pharmacy (the ePrEP Kenya Pilot; ClinicalTrials.gov: NCT05377138, May 11, 2022) that was informed by the DCE findings. We conducted both studies in partnership with MYDAWA, Kenya’s first licensed online pharmacy. MYDAWA uses an English-language site available online via smartphone or computer. At the time of this study, online delivery of PrEP/PEP services was limited to Nairobi County. Detailed protocols for these studies are published elsewhere [18, 20].

We recruited DCE participants (i.e., potential online PrEP users) via banner ads on MYDAWA webpages for HIV self-tests; interested participants called the study phone number included on banner ads to screen for eligibility. Eligible DCE participants were ≥ 18 years of age, interested in PrEP, and not known to have HIV. We recruited ePrEP Kenya pilot participants (i.e., current online PrEP/PEP users) also using banner ads on MYDAWA’s sexual wellness page; individuals interested in online PrEP/PEP services completed a self-screen tool that identified if they had any behaviors associated with HIV risk (PrEP) or HIV exposure in the past 72 h (PEP) [21]. Pilot participants self-selected into either online PrEP or PEP services based on the self-screen tool. Eligible pilot participants were ≥ 18 years of age, reported behaviors associated with HIV risk or a recent HIV exposure, and were willing to pay for online HIV testing services (HIV self-test or rapid diagnostic test with online verification of HIV status; ~$2 USD) and courier fees (~$1 USD) associated with the delivery of these services and PrEP/PEP drugs.

### Ethics

We received approvals for both studies from the Scientific and Ethics Review Unit at the Kenya Medical Research Institute (Nairobi, Kenya; KEMRI/RES/7/3/1) and Institutional Review Board at the University of Washington (Seattle, USA; STUDY00014011). All participants provided written informed consent for study activities.

### Study procedures and data collection

We completed in-person quantitative surveys with potential online PrEP users at an arranged time and convenient location. Questionnaires were administered electronically by trained Kenyan researchers using Sawtooth Software [22]. In the questionnaires, we assessed participants’ demographics, recent HIV testing behaviors, PrEP use prior to enrolling in the study, prior online pharmacy purchases, and WTP for online PrEP services. Potential online PrEP users also responded to questions about type and number of sexual partners in the prior 3 months, condomless sex, HIV exposure, and diagnosis or treatment for a sexually transmitted infection in the prior 6 months. To increase privacy while reporting sensitive sexual behaviors, we gave potential online PrEP users the option to self-enter their responses to these questions directly on the interviewer’s tablet.

We completed questionnaires with current online PrEP/PEP users after they initiated online PrEP or PEP services. To initiate online PrEP/PEP services, these participants had to complete a telehealth consultation with a remote clinician, have HIV testing services delivered to a preferred location, and have PrEP/PEP drugs delivered to a preferred location by courier. Couriers were pharmacy technicians trained in conducting HIV rapid diagnostic tests (RDT). During their telehealth consultations, participants were counseled on HIV risk, PrEP safety, and completed a medical screening assessment. For HIV testing services, participants had the option of at-home blood-based HIV self-testing (HIVST) for a subsidized fee of 250 KES (~$2 USD) or RDT conducted by the courier at a location chosen by the client for a non-subsidized fee of 150 KES (~$1 USD). Prior to any PrEP/PEP delivery, all current online PrEP/PEP users uploaded an image of their HIVST or RDT result to the online delivery platform and received confirmation of HIV-negative status from a remote MYDAWA clinician. Couriers waited with participants to ensure receipt of HIV status confirmation, and participants had the option to have PrEP/PEP drugs delivered at the same or at a later time from HIV testing services. We completed questionnaires with current online PrEP/PEP users after the first PEP or PrEP delivery. We assessed participants’ demographics, prior online pharmacy purchases, PrEP/PEP use prior to study enrollment, behaviors associated with HIV risk in the past 6 months, and WTP for online PrEP/PEP delivery. Current online PrEP/PEP users also responded to questions about their current relationship status, number of sexual partners in the prior 3 months, new sex partners in the prior 3 months, and condomless sex in the prior 2 weeks. Since the pilot study used government PrEP/PEP commodities, clients were not charged for drugs; however, a delivery fee of up to 149 KES (~$1 USD) was applied per courier visit. Differences in demographic, socioeconomic, and behavioral data collected from each cohort are summarized in Appendix Section 1 (Supplementary Tables 1–3).

### WTP data

We used a stated preference approach with direct open-ended questions about the amount potential and current online PrEP/PEP users would be willing to pay for different components of online PrEP and PEP service delivery (Appendix Section 1) [23]. Potential online PrEP users were only asked how much they would be willing to pay for online PrEP, not online PEP, services. Specifically, we asked participants the maximum price, in Kenyan shillings (KSH), they were willing to pay for different components of online PrEP delivery—i.e., a telehealth consultation with a remote clinician, at-home HIV testing services, a one-month PrEP supply, and courier delivery fees—as well as the maximum price they were willing to pay for the total package of online PrEP services. For HIV testing, we asked potential online PrEP users how much they would be willing to pay for blood-based and for oral-fluid HIVST. WTP survey questions were evaluated via cognitive interviews and pilot-testing before study launch [20].

Current online PrEP/PEP users were asked approximately how much they would be willing to pay for both online PrEP and online PEP delivery. We asked participants how much they would be willing to pay for each of the following: a telehealth consultation, blood-based HIVST, provider-administered RDT, and a three-month PrEP supply. Current online PrEP/PEP users were additionally asked how much they would be willing to pay for a 28-day course of PEP, excluding delivery fees. To inform WTP for new PrEP products, we also asked these participants how much they would be willing to pay for monthly PrEP injection and a monthly PrEP vaginal ring (female participants only), available online through MYDAWA, excluding delivery fees. We did not ask current online PrEP/PEP users about WTP for delivery fees associated with the different package components, as these are often a standard fee set across all products for online pharmacies. Both potential and current online PrEP/PEP users were instructed to enter “zero” as the amount they were willing to pay if they were not willing to pay for the service.

### Statistical analysis

We utilized descriptive statistics to characterize study populations. Our primary outcome among participants in both studies was WTP for the total package of online PrEP services. Since potential online PrEP users reported WTP for a one-month PrEP supply and current online PrEP/PEP users reported WTP for a three-month PrEP supply in their reported package of online PrEP services, we generated a metric of total WTP among current online PrEP/PEP users from their reported WTP for different components of the model, dividing WTP for a three-month PrEP supply by three to generate a comparable WTP for a one-month PrEP supply. To understand participant characteristics associated with differences in mean total WTP in each study, we used generalized linear models with a gamma distribution and log link to adjust for skewed distributions of WTP data. Variables were selected for inclusion in bivariate models based on a priori knowledge from stakeholder input, of potential associations with HIV acquisition risk, PrEP uptake, and WTP. This was done to identify demographic factors associated with differences in WTP and to determine whether characteristics associated with greater risk of HIV acquisition were associated with higher WTP. Variables were selected for inclusion in multivariable models based on significance (*p* ≤ 0.1) in bivariate models measuring association between each individual variable and WTP for PrEP or PEP. We used *p* ≤ 0.05 to determine significance in multivariable models. Models were separately fit for potential and current online PrEP/PEP users using Stata 18.0 (StataCorp LLC, College Station, TX). WTP data are reported in KSH, and converted to US Dollars using the average exchange rate from when the data were collected (May 2022 through December 2023): 118 KSH to 1 USD.

## Results

We included 772 potential online PrEP users and 740 current online PrEP/PEP users in the analysis who completed either the DCE or pilot surveys and responded to the WTP questions. In the pilot study 85% (1915/2257) of screened participants were enrolled, of whom 740 (39%) completed behavioral questionnaires including WTP questions. Among potential online PrEP users, mean age was 26.8 years, 54.2% were female, and 32.0% were enrolled in school (Table 1). Median monthly income among potential online PrEP users was 10,000 KSH ($85 USD; IQR 5,000–20,000 KSH). A substantial proportion (38.9%) reported ever having taken PrEP, and 18.5% reported currently taking PrEP at the time of the survey. When queried about sexual behavior associated with HIV acquisition in the 6 months prior to the survey, 79.7% of respondents reported having had condomless sex, 32.3% had a possible HIV exposure, and 13.7% had been diagnosed or treated for a sexually transmitted infection (STI). Among current online PrEP/PEP users, mean age was 29.2 years, most (61.5%) were male, and 25.5% were enrolled in school. Most had enrolled in the study to obtain PEP (84.3%) rather than PrEP (14.1%), and most (75.1%) reported a monthly income above 10,000 KSH ($85 USD). Few current online PrEP/PEP users (11.4%) had ever used PEP or PrEP prior to enrolling in the study, and a third (30.8%) had made online pharmacy purchases prior to the study. Overall, 73.4% of current online PrEP/PEP users reported having a new sexual partner in the prior 3 months, and 26.6% reported condomless sex in the 2 weeks prior to responding to the questionnaire. Compared to current online PrEP/PEP users, potential online PrEP users were younger, had lower monthly income, and a higher proportion were female and currently enrolled in school. Potential online PrEP users were also more likely to have used PrEP in the past, reported lower number of sexual partners, and were less likely to have made online pharmacy purchases than current online PrEP/PEP users. Characteristics of current online PrEP/PEP users (pilot participants) by PrEP vs. PEP arm at enrollment are detailed in Appendix Section 2 (Supplementary Tables 4 & 5).

**Table 1:** Characteristics of potential online PrEP Users (DCE questionnaire respondents) and current online PrEP/PEP users (pilot questionnaire respondents)

	**Potential Online PrEP Users, ***N***=772**		**Current Online PrEP/PEP Users, ***N***=740**		
	**n**	**%**	**n**	**%**	***p*-value**^**a**^
Demographic and Socioeconomic Characteristics					
Study Arm					
PEP Only	--	--	625	84.3	
PrEP Only			104	14.1	
PEP to PrEP Transition			11	1.5	
Age					
18-24	333	43.1	237	32.0	<0.001
25 or older	439	56.9	503	68.0	
Sex^b^					
Female	418	54.2	283	38.2	<0.001
Male	345	44.7	455	61.5	
Currently Enrolled in School					
No	525	68.0	551	74.5	0.006
Yes	247	32.0	189	25.5	
Employment					
Not employed	168	21.8	--	--	
Part time or seasonal employment	385	49.9			
Multiple jobs or full-time employment	210	27.2			
Monthly income					
10,000 KSH or less	348	45.1	184	24.9	<0.001
More than 10,000 KSH	323	41.8	556	75.1	
PrEP and Online Pharmacy Engagement					
Ever taken PrEP or PEP^c^					
No	449	58.2	656	88.7	<0.001
Yes	300	38.9	84	11.4	
Currently taking PrEP or PEP					
No	606	78.5	0	0.0	<0.001
Yes	143	18.5	740	100.0	
Ever purchased products from an online pharmacy					
No	582	75.4	509	68.8	0.006
Yes	190	24.6	228	30.8	
Sexual Behavior					
Type of sexual partners, prior 3 months					
Did not have sex	85	11.0	--	--	
1 primary partner and no other partners	269	34.8			
1 primary and 1+ casual partner(s)	238	30.8			
1+ casual partner(s)	172	22.3			
Current relationship status					
Primary partner only	--	--	253	34.2	
Casual partners only			370	50.0	
Primary and casual partners			92	2.4	
Number of sexual partners in prior 3 months					
0^d^	133	17.2	6	0.8	<0.001
1	232	30.1	308	41.6	
2	165	21.4	284	38.4	
3	87	11.3	71	9.6	
4 or more	126	16.3	59	8.0	
Sex without a condom, prior 6 months					
No	155	20.1	--	--	
Yes	615	79.7			
Exposure to HIV, prior 6 months					
No	380	49.2	--	--	
Yes	249	32.3			
Unsure	143	18.5			
Diagnosis or treatment of STI, prior 6 months					
No	664	86.0	--	--	
Yes	106	13.7			
New sexual partner in prior 3 months					
No	--	--	179	24.2	
Yes			543	73.4	
Sexual intercourse in prior 2 weeks					
No	--	--	378	51.1	
Yes			355	48.0	
Condomless sexual intercourse in prior 2 weeks					
No	--	--	536	72.4	
Yes			197	26.6	

*KSH* Kenyan shillings, *PEP* post-exposure prophylaxis, *PrEP* pre-exposure prophylaxis, *SD* standard deviation, *STI* sexually transmitted infection, *WTP* willingness to pay

^a^*p*-value for comparison between Potential online PrEP users vs. current online PrEP/PEP users

^b^Due to missing values, some percentages may not sum to 100

^c^ Potential online PrEP users were asked about PrEP use prior to entering the study; Current online PrEP/PEP users were asked about PEP or PrEP use prior to entering the study

^d^48 participants included in this category reported having had sex in the prior 3 months, but also reported zero sexual partners. The remaining 85 participants reported not having had sex in the prior 3 months

On average, potential online PrEP users were willing to pay a maximum of 1388.4 KSH ($11.77 USD) for the package of PrEP delivery services, while current online PrEP/PEP users were willing to pay approximately 1271.2 KSH ($10.77 USD; Table 2). Seven potential online PrEP users (0.9%) and nineteen current online PrEP/PEP users (2.6%) reported zero as the amount they were willing to pay for PrEP services. Among potential online PrEP users, mean maximum WTP for each component of PrEP service delivery ranged from 192.9 KSH ($1.63 USD) for pill delivery to 480.8 KSH ($4.07 USD) for a one-month supply of PrEP. Among current online PrEP/PEP users, mean WTP ranged from 269.1 KSH ($2.28 USD) for a blood-based self-test to 506.3 KSH ($4.29 USD) for a one-month supply of PrEP. Current online PrEP/PEP users were willing to pay more than potential online PrEP users for both the remote clinical consultation (495.7 vs. 362.1 KSH, *p* < 0.001) and the blood-based self-test (269.1 vs. 242.6 KSH, *p* = 0.01). Current online PrEP/PEP users were willing to pay 1514.7 KSH ($12.84 USD) for monthly injectable PrEP, and 767.7 KSH ($6.51 USD) for monthly PrEP vaginal ring (Table 2). WTP by pilot arm at enrollment (PrEP vs. PEP) are detailed in Appendix Section 2; there were no differences in WTP by arm at enrollment. A complete summary of WTP responses is in Appendix Section 3 (Supplementary Tables 6 & 7; Supplementary Figs. 1–15).

**Table 2:** Willingness to pay (KSH)^a^ for components of online pharmacy PrEP delivery, among potential online PrEP users and current online PrEP/PEP users

	**Potential Online PrEP Users** **(*N*=772)**		**Current Online PrEP/PEP Users** **(*N*=740)**		
	**Median (IQR)**	**Mean (SD)**	**Median (IQR)**	**Mean (SD)**	***p*-value**^**d**^
Package of online PrEP/PEP services^b^					
Online PrEP services: 1-month supply	1000 (800-1975)	1388.41 (995.74)	1000 (567 – 1667)	1271.16 (1340.38)	0.05
Online PEP services: 28-day supply	--	--	500 (200 – 1000)	812.95 (1376.61)	--
Package components					
Telehealth visits with remote clinician	300 (150-500)	362.09 (480.06)	400 (125-500)	495.74 (667.65)	<0.001
Blood-based HIV self-test	200 (100-300)	242.62 (182.66)	250 (150-300)	269.11 (214.09)	0.01
Oral HIV self-test	200 (100-300)	231.72 (160.79)	--	--	--
PrEP drugs: one-month supply^c^	300 (200-500)	480.83 (550.89)	333 (167 – 667)	506.30 (656.52)	0.4
PrEP drugs: three-month supply^c^			1000 (500 – 2000)	1518.91 (1969.57)	--
Delivery of PrEP pills	200 (100-200)	192.85 (120.88)	--	--	--
New PrEP products					
Monthly injectable^e^	--	--	1000 (500 – 2000)	1514.73 (2671.17)	
Monthly vaginal ring^f^	--	--	500 (250 – 1000)	767.74 (727.16)	

^a^Average USD/KSH 2022 exchange rate = 118 KSH per 1 USD

^b^Package of PrEP delivery services includes HIV testing, remote clinical consultation, 1-month supply of PrEP medication, and delivery

^c^Excluding delivery fees

^d^*p*-value for comparison of means using ANOVA

^e^*n*=554 participants completed questions about WTP for monthly injectable PrEP

^f^*n*=31 participants completed questions about WTP for vaginal ring

In bivariate models, of potential online PrEP users, male sex, being enrolled in school, being employed (either full-time or at multiple jobs), higher monthly income, and history of online pharmacy purchases were associated with higher mean WTP (Supplementary Tables 8 & 10). Prior PrEP use and having 4 or more sexual partners were associated with lower mean WTP. Other sexual behaviors were not significantly associated with WTP. In the multivariable model for potential online PrEP users, male sex, being enrolled in school, having higher income, and history of online pharmacy purchases remained associated with higher WTP (Table 3). Respondents with a history of online pharmacy purchases were willing to pay 23% more than those without a history of online pharmacy purchases (1.23; 95% CI: 1.09–1.39). Potential online PrEP users aged 25 or older were not willing to pay as much as younger participants (0.88; 95% CI: 0.76–0.95), and prior PrEP users were willing to pay 28% less than respondents with no history of PrEP use (0.72; 95% CI: 0.65–0.85). For potential online PrEP users, employment status and number of recent sexual partners were no longer significantly associated with mean WTP. Among current online PrEP/PEP users, in bivariate models, older age, higher monthly income, history of online pharmacy purchases, recent sex and recent condomless sex were associated with higher mean WTP (Supplementary Tables 9 & 10). Current enrollment in school was associated with lower mean WTP. In the multivariable model for current online PrEP/PEP users, higher monthly income and prior online pharmacy purchases remained associated with higher mean WTP. Participants with higher monthly income were willing to pay 43% more than participants with monthly income 10,000 KSH or lower (1.43; 95% CI: 1.19–1.73), and those with a history of online pharmacy purchases were willing to pay 19% more (1.19; 95% CI: 1.03–1.36). Differences in sexual behavior, age, and school enrollment were no longer associated with mean WTP after adjusting for other demographic and behavioral characteristics.

**Table 3:** Characteristics associated with differences in mean WTP^a^ for the package of PrEP delivery services (HIV testing, remote clinical consultation, 1-month supply of PrEP) among potential online PrEP users and current online PrEP/PEP users; multivariable models

	**Potential online PrEP users;** ***N*=772**			**Current online PrEP/PEP users;** ***N*=740**		
	**Adj. Coef.**^**b,c**^	**95% CI**	***p*-value**	**Adj. Coef.**^**d**^	**95% CI**	***p*-value**
Demographic and socioeconomic characteristics						
Age						
18-24	Ref.	--	--	Ref.	--	--
25 or older	0.88	0.76 – 0.95	0.04	1.18	0.99 – 1.39	0.06
Sex						
Female	Ref.	--	--			
Male	1.17	1.07 – 1.32	0.004			
Currently Enrolled in School						
No	Ref.	--	--	Ref.	--	--
Yes	1.14	1.01 – 1.29	0.03	1.09	0.91 – 1.29	0.4
Employment						
Not employed	Ref.	--	--			
Part time or seasonal employment	1.08	0.92 – 1.22	0.3			
Multiple jobs or full time employment	1.10	0.87 – 1.26	0.3			
Monthly income						
10,000 KSH or less	Ref.	--	--	Ref.	--	--
More than 10,000 KSH	1.25	1.10 – 1.43	0.001	1.43	1.19 – 1.73	<0.001
PrEP and Online Pharmacy Engagement						
Ever taken PrEP						
No	Ref.	--	--			
Yes	0.72	0.63 – 0.80	<0.001			
Ever purchased products from an online pharmacy						
No	Ref.	--	--	Ref.	--	--
Yes	1.23	1.09 – 1.40	0.001	1.19	1.03 – 1.36	0.02
Sexual Behavior						
Current relationship status						
Primary partner only				Ref.	--	--
Casual partners only				0.88	0.76 – 1.02	0.09
Primary and casual partners				0.84	0.69 – 1.04	0.1
Number of sexual partners, prior 3 months						
0	Ref.	--	--			
1	1.08	0.93 – 1.26	0.3			
2	1.00	0.85 – 1.17	1.0			
3	1.03	0.85 – 1.26	0.8			
4 or more	1.02	0.85 – 1.23	0.8			
Sexual intercourse in prior 2 weeks						
No				Ref.	--	--
Yes				1.10	0.93 – 1.29	0.3
Condomless sexual intercourse in prior 2 weeks						
No				Ref.	--	--
Yes				1.03	0.85 – 1.24	0.8

*KSH* Kenyan shillings, *PEP* post exposure prophylaxis, *PrEP* pre-exposure prophylaxis, *SD* standard deviation, *STI* sexually transmitted infection, *WTP* willingness to pay

^a^WTP refers to maximum willingness to pay, in Kenyan shillings, for the total package of PrEP delivery services including HIV testing, remote clinical consultation, PrEP medication, and delivery fees

^b^All coefficients have been exponentiated

^c^Model adjusted for age, sex, current school, employment type, income, prior PrEP use, prior e-pharmacy use, number of sex partners in prior 3 months. Constant for multivariable model = 1152.8 KSH

^d^Model adjusted for age, current school, income, prior e-pharmacy use, relationship type, recent sexual intercourse, recent condomless sexual intercourse. Constant for multivariable model = 829.7 KSH

Current online PrEP/PEP users were willing to pay an average of 812.9 KSH ($6.89 USD) for a 28-day supply of PEP pills, and 138 (18.6%) reported zero as the amount they were willing to pay. Among current online PrEP/PEP users, in bivariate models, being age 25 or older, not being enrolled in school, higher income, and history of online pharmacy purchases were associated with higher WTP for a 28-day course of PEP (Table 4). However, in the adjusted model, only age and history of online pharmacy purchases were associated with differences in WTP. Current online PrEP/PEP users aged 25 or older were willing to pay 34% more than participants age 18–24 (1.34; 95% CI: 1.02–1.75), and participants who had made online pharmacy purchases in the past were willing to pay 42% more than those who had not (1.42; 95% CI: 1.13–1.78). There were no measured characteristics associated with WTP = 0.=

**Table 4:** Characteristics associated with differences in mean WTP^a^ for a 28-day course of PEP among current online PrEP/PEP users (*N*=740); multivariable models

	**Coef.**	**95% CI**	**p-value**	**Adj. Coef.**^**b,c**^	**95% CI**	***p*-value**
Demographic and Socioeconomic Characteristics						
Pilot Arm						
PEP Only	Ref.	--	--			
PrEP Only	1.09	0.77 – 1.55	0.6			
PEP to PrEP Transition	0.77	0.28 – 2.11	0.6			
Age						
18-24	Ref.	--	--	Ref.	--	--
25 or older	1.55	1.20 – 1.99	0.001	1.34	1.02 – 1.75	0.03
Sex						
Female	Ref.	--	--			
Male	1.15	0.90 – 1.47	0.3			
Currently Enrolled in School						
No	Ref.	--	--	Ref.	--	--
Yes	0.76	0.58 – 0.99	0.04	0.98	0.73 – 1.29	0.9
Monthly income						
10,000 KSH or less	Ref.	--	--	Ref.	--	--
More than 10,000 KSH	1.64	1.26 – 2.14	<0.001	1.28	0.93 – 1.74	0.1
PrEP and Online Pharmacy Engagement						
Ever taken PrEP or PEP						
No	Ref.	--	--			
Yes	0.96	0.66 – 1.42	0.9			
Ever purchased products from an online pharmacy						
No	Ref.	--	--	Ref,	--	--
Yes	1.50	1.19 – 1.89	0.001	1.42	1.13 – 1.78	0.002
Sexual Behavior						
Current relationship status						
Primary partner only	Ref.	--	--			
Casual partners only	0.87	0.66 – 1.14	0.3			
Primary and casual partners	1.01	0.67 – 1.53	1.0			
Number of sexual partners, prior 3 months						
0	Ref.	--	--			
1	1.64	0.48 – 5.63	0.4			
2	1.73	0.50 – 5.96	0.4			
3	1.80	0.50 – 6.43	0.4			
4 or more	2.49	0.69 – 8.99	0.2			
New sexual partner in prior 3 months						
No	Ref.	--	--			
Yes	1.08	0.82 – 1.44	0.6			
Sexual intercourse in prior 2 weeks						
No	Ref.	--	--			
Yes	1.20	0.95	0.1			
Condomless sexual intercourse in prior 2 weeks						
No	Ref.	--	--			
Yes	1.19	0.90 – 1.57	0.2			

*KSH* Kenyan shillings, *PEP* post-exposure prophylaxis, *PrEP* pre-exposure prophylaxis, *WTP* willingness to pay

^a^WTP refers to maximum willingness to pay, in Kenyan shillings, for a 28-day course of PEP pills, excluding delivery fees

^b^All coefficients have been exponentiated

^c^Model adjusted for age, current school, income, prior e-pharmacy use. Constant for multivariable model = 481.1 KSH

## Discussion

We assessed stated WTP among both current and potential online PrEP/PEP users in Kenya along with characteristics associated with WTP. Potential and current online PrEP/PEP users were willing to pay the same median amount for package of online PrEP services and also reported similar WTP for blood-based HIVST and PrEP drugs. However, comparisons of WTP should take into account that different questions were asked in each group; potential online PrEP users were asked about their maximum WTP while current online PrEP/PEP users were asked about approximate WTP. Therefore, WTP for current online PrEP/PEP users likely underestimates their maximum WTP. Despite this caveat, knowing a price point at which both current and potential users are willing to pay (whether maximum or average) can inform service pricing to maximize coverage of ePrEP services.

Potential online PrEP users who were PrEP naïve had a higher WTP than those with a history of prior PrEP use, likely since prior PrEP users had successfully overcome barriers associated with clinic-based PrEP (i.e. long wait times, limited hours of operation, stigma) to obtain it free of charge. This finding is consistent with a DCE conducted by our team on the same participants which showed a stronger preference for online PrEP among PrEP naïve participants [24]. Taken together, these results suggest that online PrEP could expand PrEP coverage to interested individuals whose preferences are not met by standard PrEP services. Current PrEP use is low among those who can benefit; therefore acceptable and sustainable strategies are needed to expand coverage to those at risk of HIV who experience barriers to clinic-based PrEP.

In both study groups, higher monthly income and history of purchases via the online pharmacy were associated with willingness to pay a higher amount for PrEP services, likely due to higher disposable income. There were trends in opposite directions for age between the two groups, with potential online PrEP users, age 25 and older willing to pay less than those aged 18–24. However, older current online PrEP/PEP users willing to pay more than younger participants. Notably, almost all participants were willing to pay at least some amount for online-delivered PrEP (99.1% of potential users and 97.4% of PrEP/PEP users). Among current online PrEP/PEP users, older age and history of online pharmacy purchases were associated with higher WTP for PEP. Overall, our results suggest there is a demand for PrEP and PEP delivery via online pharmacies, and willingness to pay for these services.

Despite differences in demographics and question wording, the two study groups had similar WTP for PrEP, which suggests that WTP may be comparable among potential and current users of online pharmacy-based HIV prevention. Current online PrEP/PEP users were older than potential online PrEP users and had higher monthly income. Potential online PrEP users were interested in PrEP; however, most were not using it at the time of the questionnaire and none were currently obtaining online pharmacy PrEP. A higher proportion of potential online PrEP users had taken PrEP or PEP in the past compared to current online PrEP/PEP users. Current online PrEP/PEP users responded to the questionnaire after initiating PEP or PrEP and having paid for their HIV self-test and delivery of PEP or PrEP via the online pharmacy; therefore, responses to WTP questions may have been influenced by the amount they paid for the service, potentially resulting in downward bias. Additionally, most current online PrEP/PEP users (> 80%) were PEP clients, and few of them transitioned to PrEP after completing their PEP regimen, possibly indicating a difference in interest in PrEP across participant groups. Despite these differences, mean WTP for PrEP differed by only 117 KSH ($1 USD) between PEP vs. PrEP users and median WTP was the same.

To our knowledge, there are no prior studies evaluating WTP for PrEP and PEP service delivery via online pharmacies in Africa and no data on PEP WTP. However, there are limited data on individuals’ WTP for brick-and-mortar pharmacy-delivered PrEP to contextualize our findings. A recent qualitative study among adolescent girls and young women (AGYW) accessing PrEP through retail pharmacies in Kenya found over half (54%) of participants reported they were willing to pay for PrEP via retail pharmacies even if it could be obtained for free at clinics, due to convenience of locations, lack of stigma, and lack or queues or medication stock outs [12]. Similarly, in a population-level survey among 18–34-year-olds in western Kenya, 61% of respondents with PrEP awareness indicated they were willing to pay for PrEP [25]. In a cross-sectional survey among female sex workers and men who have sex with men in Nigeria, researchers used a bidding game iteration process to estimate the amount participants were willing to pay for PrEP services. Most participants (73%) indicated they were willing to pay for PrEP services; 17% were willing to pay the market price for PrEP (N3,500; $9.20 USD 2020), and a further 36% were willing to pay if PrEP cost between N2,500 and N3,400 ($6.60–9.00 USD 2020). In multivariate models, there were no measured factors associated with WTP (including employment, monthly income, age, or marital status) [26]. In contrast to these studies, we found almost all participants were willing to pay at least some amount for PrEP services and they had a slightly higher WTP; this may be due to participants in our studies having higher socio-economic status that those of previous studies. For example, while participants in the Nigerian study had a median monthly income of $39 USD, most participants (42% of potential online PrEP users and 75% of current online PrEP/PEP users) reported a monthly income of 10,000 KSH ($85 USD) or higher. In a population-level survey from Western Kenya, 48% of respondents with PrEP awareness reported the maximum they were willing to pay for a 1-month supply was between 100 and 499 KSH (~$1-$5 USD) [25]. An additional17% were willing to pay 500–1000 KSH, and 30% were willing to pay < 100 KSH with WTP greater among men than women. We found potential and current online PrEP/PEP users were willing to pay a median 300–333 KSH ($2.54-$2.82 USD) for a one-month supply of PrEP. We similarly observed that men were willing to pay more for PrEP services than women, although only among potential online PrEP users. An important caveat of online pharmacy PrEP/PEP is that it largely reach persons with higher socioeconomic status and internet access/literacy. While this platform can expand PrEP coverage to Kenya’s growing middle class, other PrEP/PEP provision strategies are needed to reach those with lower income or digital literacy.

Our results should be interpreted in light of several limitations. First, WTP questions differed between the two questionnaires, and differences in question wording and content impacted our ability to draw direct comparisons between the study groups. For example, current online PrEP/PEP users were asked about WTP for a three-month supply of PrEP drugs, while potential online PrEP users were asked about WTP for one month. To facilitate comparisons between the groups, we divided the former WTP by 3 to estimate WTP for one month of PrEP. However, participants might be WTP more for the greater convenience of a 3-month supply of PrEP, which would overestimate their one-month WTP. Despite this, estimates of median WTP for one month of PrEP were similar between the two groups. Secondly, questions about sociodemographic and behavioral characteristics, including questions about sexual behavior, differed between the questionnaires, limiting our ability to compare the associations of some characteristics with WTP between potential and current online PrEP/PEP users. Additionally, recruitment of potential online PrEP users relied on an opt-in approach in which individuals contacted the study to complete an in-person survey; individuals with greater privacy concerns may be underrepresented. Further, most participants in the pilot study were PEP users, who may have lower interest in PrEP use. Further, we measure stated WTP, which may not accurately reflect individuals’ real world uptake; future studies should measure observed WTP. Finally, to reduce participant time and staff burden in completing WTP questions, we utilized an open-ended question approach. Although commonly utilized in the HIV literature [27–29], this approach does not incorporate more nuanced WTP methods such as iterative approaches (e.g. bidding games) that raise and lower proposed prices until the participants highest WTP is estimated [30, 31]. Future studies are needed that further elicit participant’s WTP using more comprehensive methods.

Strengths of our study include a large sample size and ability to compare results from actual vs. potential online PrEP users recruited from separate studies. Evaluating WTP among both potential online PrEP/PEP users increasing the generalizability of our findings beyond WTP of persons enrolled in a pilot study of online PrEP/PEP services. Additionally, pilot study participants were screened for HIV risk indication and willingness to obtain PrEP/PEP from online pharmacies. Similarly, all potential online PrEP users reported interest in online PrEP/PEP and most reported recent behaviors associated with HIV risk including multiple partners, known exposure to HIV, or treatment for an STI. Recruitment strategies for both groups via MYDAWA’s website ensured that participants had sufficient computer literacy to obtain PrEP/PEP online. To our knowledge, this was the first study to measure the amount potential and current online PrEP and PEP users are willing to pay to access services via online pharmacies.

## Conclusion

We observed a demand and willingness to pay for PrEP and PEP services via online pharmacies among eligible individuals in Kenya. Expanding PEP and PrEP services through telehealth platforms will likely improve access to these services, particularly for individuals not accessing clinic-based PrEP/PEP. Our results can inform the design of public/private partnerships between governments and online retail pharmacies to deliver PrEP/PEP services at appropriate price points to reduce HIV burden.

## Supplementary Information

Supplementary Material 1.

## Data Availability

The data generated and analyzed during the study are not publicly available due to privacy and ethical restrictions, but are available from the corresponding author on reasonable request.
