# Supplementary material for: Willingness to pay for HIV pre- and post-exposure prophylaxis services delivered via an online pharmacy in Kenya
Source: BMC Health Serv Res. 2025 Apr 22;25:576. doi: 10.1186/s12913-025-12766-x (PMC12013023; doi:10.1186/s12913-025-12766-x)
Supplement: Supplementary file 1 — Supplementary Material 1. [file 12913_2025_12766_MOESM1_ESM.docx]

**Supplemental Appendix for the manuscript: “Willingness to pay for HIV pre- and post-exposure prophylaxis services delivered via an online pharmacy in Kenya”**

**Appendix Section 1. Summary of demographic, socioeconomic, behavioral, and WTP data collected from potential online PrEP users (DCE participants) and current online PrEP/PEP users (pilot participants)**

| Supplementary Table 1. Summary of differences in data collected in questionnaires administered to potential online PrEP users and current online PrEP/PEP users | | |
| --- | --- | --- |
|  | DCE (Potential PrEP Users) | Pilot (Current PrEP/PEP Users) |
| Demographic and Socioeconomic Data | Age  Sex  Enrollment in school (current)  Monthly income  Employment (current) | Age  Sex  Enrollment in school (current)  Monthly income |
| PrEP/PEP Use and Online Pharmacy Engagement | Ever taken PrEP prior to study enrollment  Currently taking PrEP  Ever purchased products from online pharmacy prior to study enrollment | Ever taken PrEP/PEP prior to study enrollment  Currently taking PrEP/PEP  Ever purchased products from online pharmacy prior to study enrollment |
| Sexual Behavior | Type of sexual partners, prior 3 months  Number of sexual partners in prior 3 months  Sex without a condom, prior 6 months  Exposure to HIV, prior 6 months  Diagnosis or treatment of STI, prior 6 months | Current relationship status  New sexual partner in prior 3 months  Sexual intercourse in prior 2 weeks  Condomless sexual intercourse in prior 2 weeks |
| WTP | Maximum WTP for each component of online PrEP service, and total package of online PrEP services (detailed questions below) | Approximate and maximum WTP for each component of online PrEP service, PEP service, new PrEP technologies (detailed questions below) |

| Supplementary Table 2. Willingness to pay questions from DCE questionnaire, administered to potential online PrEP users | |
| --- | --- |
| The next set of questions will ask about how much you would like to pay for different parts of online PrEP delivery. For each part, please provide the maximum price you are willing to pay. In answering these questions, please consider your usual expenses. Remember that there are no right or wrong answers. We’re interested in your preference. You can write zero if you feel that most accurately reflects your preference. | |
| What is the maximum price you are willing to pay for a blood-based HIV self-test? | _________ KSH |
| What is the maximum price you are willing to pay for an oral HIV self-test? | _________ KSH |
| What is the maximum price you are willing to pay for remote/online clinical consultation to obtain a prescription for PrEP based on your HIV self-test results (one-time cost)? | _________ KSH |
| What is the maximum price you are willing to pay for a one-month supply of PrEP medicines? | _________ KSH |
| What is the maximum price you are willing to pay for delivery of PrEP medicines to a setting of your choice (one-time courier fee)? | _________ KSH |
| What is the maximum price you are willing to pay in total for the package of services associated online PrEP delivery (this includes courier delivery of an HIV self-test, a remote clinical consultation, and courier-delivered PrEP)? | _________ KSH |

| Supplementary Table 3. Willingness to pay questions from pilot questionnaire, administered to current online PrEP/PEP users | |
| --- | --- |
| We are interested in learning about your willingness to pay for services and products associated with online HIV prevention outside this study.  The following questions are hypothetical, and you will not be asked to pay these amounts for any services provided in this study. In answering these questions, please consider your usual income and expenses in healthcare. | |
| The last time you went out to eat, how much money did you spend? (in KSH) | _________ KSH |
| Imagine you are interested in getting HIV pre-exposure prophylaxis, PrEP, through an online pharmacy outside of this trial. To get PrEP this way, you will need to complete blood-based HIV self-test delivered by a courier to a setting of your choice and upload an image of the test to a secure online system. | |
| Approximately how much would you be willing to pay for online-ordered blood-based HIV self-test to start PrEP, not including delivery fees?  If you are not willing to pay anything, you can say 0 (zero). | _________ KSH |
| If you had to pay this amount right now to receive the product, how sure are you that you would pay this? | Totally sure  Somewhat sure  Neither sure nor unsure  Not very sure  Unsure  Prefer not to answer |
| Above which price would you definitely not buy an online blood-based HIV self-test to start PrEP? | _________ KSH |
| In order to get PrEP from an online pharmacy, you would need to have a remote/online clinical consultation with a medical provider to make sure PrEP is safe for you to use. This can be done over the phone or a video chat and would last approximately 30 minutes | |
| Approximately how much would you be willing to pay for a remote/online clinical encounter to start PrEP?  If you are not willing to pay anything, you can say 0 (zero) | _________ KSH |
| If you had to pay this amount right now to receive this service, how sure are you that you would pay this? | Totally sure  Somewhat sure  Neither sure nor unsure  Not very sure  Unsure  Prefer not to answer |
| Above which price would you definitely not pay for an online/remote encounter to start PrEP? | _________ KSH |
| As part of online Pharmacy PrEP delivery, you would get a 3-month supply of PrEP drugs delivered to you: | |
| Approximately how much would you be willing to pay for a 3-month supply of PrEP drugs excluding the delivery fee?  If you are not willing to pay anything, you can say 0 (zero) | _________ KSH |
| If you had to pay this amount right now to receive the product, how sure are you that you would pay this? | Totally sure  Somewhat sure  Neither sure nor unsure  Not very sure  Unsure  Prefer not to answer |
| Instead of taking daily oral PrEP pills, imagine you could get a PrEP injection, which is a shot you get in the buttock region that would give you complete HIV protection for two months. Also, imagine you can order this injection online from MYDAWA online platform and a MYDAWA provider will meet you in a convenient location to give you this shot. | |
| Approximately how much would you be willing to pay for one PrEP injection delivered through MYDAWA, excluding delivery fees?  If you are not willing to pay anything, you can say 0 (zero) | _________ KSH |
| Instead of taking daily oral PrEP pills, imagine that you could use a PrEP vaginal ring. This is a flexible silicone ring inserted into your vagina that can reducing your risk of getting HIV by 50% but not 100%. This protection last for one month. Also, imagine you could order this ring from MYDAWA and have it delivered to you. | |
| Approximately how much would you be willing to pay for a PrEP vaginal ring delivered by MYDAWA, not including delivery fees?  If you are not willing to pay anything, you can say 0 (zero) | _________ KSH |
| The next questions are about post-exposure prophylaxis (PEP)  [INTERVIEWER if they are not PEP participants please explain:  Which means taking medicine to prevent HIV after a possible exposure. PEP is a 28-day course of daily oral medicine that must be started within 3 days after a possible exposure to HIV. ]  Imagine that you think you may have been recently exposed to HIV and could get a 28-day course of PEP delivered to you: | |
| Approximately how much would you be willing to pay to receive a 28-day course of PEP drugs, excluding delivery fees?  If you are not willing to pay any amount, you can say 0 (zero) | _________ KSH |
| Above which price would you definitely not buy an online/internet supply of PEP, excluding delivery fees? | _________ KSH |
| Health-related expenses: | |
| In the last 3 months, how much money did you spend on healthcare (e.g. including clinic visit, hospitalization, emergency care visit, not including medicines/drugs) | _________ KSH |

**Appendix Section 2. Participant characteristics and WTP by pilot study product selection at enrollment (current online PrEP users vs current online PEP users)**

| **Supplementary Table 4. Characteristics of Current Online PrEP/PEP Users by Product Selection** | | | | |  |
| --- | --- | --- | --- | --- | --- |
|  | **Online PrEP**  **n=104** | | **Online PEP**  **n=636** | |  |
|  | **n** | **%** | **n** | **%** | **p-value^a^** |
| **Demographic and Socioeconomic Characteristics** |  |  |  |  |  |
| Age  18-24  25 or older | 37  67 | 35.6  64.4 | 200  436 | 31.4  68.6 | 0.4 |
| Sex^b^  Female  Male | 37  65 | 35.6  62.5 | 246  390 | 38.7  61.3 | 0.002 |
| Currently Enrolled in School  No  Yes | 66  38 | 63.5  36.5 | 485  151 | 76.3  23.7 | 0.006 |
| Monthly income  10,000 KSH or less  More than 10,000 KSH | 36  68 | 34.6  65.4 | 148  488 | 23.3  76.7 | 0.01 |
| **PrEP and Online Pharmacy Engagement** |  |  |  |  |  |
| Ever taken PrEP or PEP^c^  No  Yes | 89  15 | 85.6  14.4 | 567  69 | 89.2  10.8 | 0.3 |
| Ever purchased products from an online pharmacy  No  Yes | 62  42 | 59.6  40.4 | 447  186 | 70.3  29.2 | 0.02 |
| **Sexual Behavior** |  |  |  |  |  |
| Current relationship status  Primary partner only  Casual partners only  Primary and casual partners | 32  49  23 | 30.8  47.1  22.1 | 221  321  69 | 34.7  50.5  10.8 | 0.003 |
| Number of sexual partners in prior 3 months  0  1  2  3  4 or more | 2  43  27  14  16 | 1.9  41.3  26.0  13.5  15.4 | 4  265  257  57  43 | 0.6  41.7  40.4  9.0  6.8 | 0.002 |
| New sexual partner in prior 3 months  No  Yes | 45  55 | 43.3  52.9 | 134  488 | 21.1  76.7 | <0.001 |
| Sexual intercourse in prior 2 weeks  No  Yes | 33  70 | 31.7  67.3 | 345  285 | 21.1  76.7 | <0.001 |
| Condomless sexual intercourse in prior 2 weeks  No  Yes | 52  51 | 50.0  49.0 | 484  146 | 76.1  23.0 | <0.001 |
| KSH: Kenyan shillings; PEP: post-exposure prophylaxis; PrEP: pre-exposure prophylaxis; SD: standard deviation  ^a^ p-value for comparison between PrEP and PEP pilot participants  ^b^ Due to missing values, some percentages may not sum to 100 | | | | |  |

| **Supplementary Table 5. Willingness to pay (KSH)^a^ for components of online pharmacy PrEP/PEP delivery, among pilot participants enrolled into online PrEP vs. PEP** | | | | | |
| --- | --- | --- | --- | --- | --- |
|  | **Online PrEP**  **n=104** | | **Online PEP**  **n=636** | |  |
|  | **Median (IQR)** | **Mean (SD)** | **Median (IQR)** | **Mean (SD)** | **p-value^c^** |
| **Package of online PrEP/PEP services** |  |  |  |  |  |
| Online PrEP services: 1-month supply | 833 (400-1683) | 1373.88 (2521.71) | 1033 (600-1666) | 1254.36 (1028.30) | 0.4 |
| Online PEP services: 28-day course | 500 (200-1000) | 878.37 (1324.43) | 500 (200-1000) | 802.25 (1385.67) | 0.6 |
| **Package components** |  |  |  |  |  |
| Telehealth visits with remote clinician | 300 (100-500) | 533.17 (1227.37) | 500 (200-500) | 489.62 (523.59) | 0.5 |
| Blood-based HIV self-test | 250 (150-300) | 285.10 (321.70) | 250 (150-300) | 266.50 (191.06) | 0.4 |
| PrEP drugs: three-month supply | 1000 (100-2250) | 1666.83 (3234.11) | 1000 (500-2000) | 1494.72 (1677.44) | 0.4 |
| **New PrEP products** |  |  |  |  |  |
| Monthly injectable^d^ | 1000 (500-2000) | 1646.69 (2477.45) | 1000 (500-2000) | 1489.81 (2707.98) | 0.6 |
| Monthly vaginal ring^e^ | 100 (0-1000) | 366.67 (550.76) | 500 (300-1000) | 810.71 (738.41) | 0.3 |
| ^a^ Average USD/KSH 2022 exchange rate = 118 KSH per 1 USD  ^b^ Summation of components of PrEP delivery services includes HIV testing, remote clinical consultation, 1-month supply of PrEP medication, and delivery  ^c^ p-value for comparison of means using ANOVA  ^d^ n=554 participants completed questions about WTP for monthly injectable PrEP  ^e^ n=31 participants completed questions about WTP for vaginal ring | | | | | |

**Appendix Section 3. Summary of WTP responses from pilot and DCE questionnaires**

| **Supplementary Table 6. Summary of WTP responses from pilot questionnaire (current PrEP/PEP users)** | | |
| --- | --- | --- |
|  | **Pilot participants**  **(N=740)** | |
| **WTP Question** | **Median (IQR)** | **Mean (SD)** |
| The last time you went out to eat, how much money did you spend? | 500 (200-1200) | 1112.04 (3921.62) |
| Approximately how much would you be willing to pay for online-ordered blood-based HIV self-test to start PrEP, not including delivery fees? | 250 (150-300) | 269.11 (214.09) |
| If you had to pay this amount right now to receive the product, how sure are you that you would pay this?  Totally sure  Somewhat sure  Neither sure nor unsure  Not very sure  Unsure | *n (%)^a^*  650 (87.8)  21 (2.8)  1 (0.1)  20 (2.7)  5 (0.7) |  |
| Above which price would you definitely not buy an online blood-based HIV self-test to start PrEP? | 500 (300-600) | 753.14 (3825.72) |
| Approximately how much would you be willing to pay for a remote/online clinical encounter to start PrEP? | 400 (125-500) | 495.74 (667.65) |
| If you had to pay this amount right now to receive this service, how sure are you that you would pay this?  Totally sure  Somewhat sure  Neither sure nor unsure  Not very sure  Unsure | *n (%)^a^*  538 (72.7)  36 (4.9)  1 (0.1)  37 (5.0)  6 (0.8) |  |
| Above which price would you definitely not pay for an online/remote encounter to start PrEP? | 800 (500-1200) | 1121.32 (1355.62) |
| Approximately how much would you be willing to pay for a 3-month supply of PrEP drugs excluding the delivery fee? | 1000 (500-2000) | 1518.91 (1969.57) |
| If you had to pay this amount right now to receive the product, how sure are you that you would pay this?  Totally sure  Somewhat sure  Neither sure nor unsure  Not very sure  Unsure | *n (%)^a^*  607 (82.0)  49 (6.6)  7 (1.0)  43 (5.8)  9 (1.2) |  |
| Approximately how much would you be willing to pay for one PrEP injection delivered through MYDAWA, excluding delivery fees?^b^ | 1000 (500-2000) | 1514.73 (2671.17) |
| Approximately how much would you be willing to pay for a PrEP vaginal ring delivered by MYDAWA, not including delivery fees?^c^ | 500 (250-1000) | 767.74 (727.16) |
| Approximately how much would you be willing to pay to receive a 28-day course of PEP drugs, excluding delivery fees? | 500 (200-1000) | 812.95 (1376.61) |
| Above which price would you definitely not buy an online/internet supply of PEP, excluding delivery fees? | 1000 (500-2000) | 1944.56 (4656.99) |
| In the last 3 months, how much money did you spend on healthcare (e.g. including clinic visit, hospitalization, emergency care visit, not including medicines/drugs) | 0 (0-1000) | 18,244.21 (442,035.50) |
| ^a^ Percentages do not sum to 100 due to nonresponse  ^b^ n=554 participants completed questions about WTP for monthly injectable PrEP  ^c^ n=31 participants completed questions about WTP for vaginal ring | | |


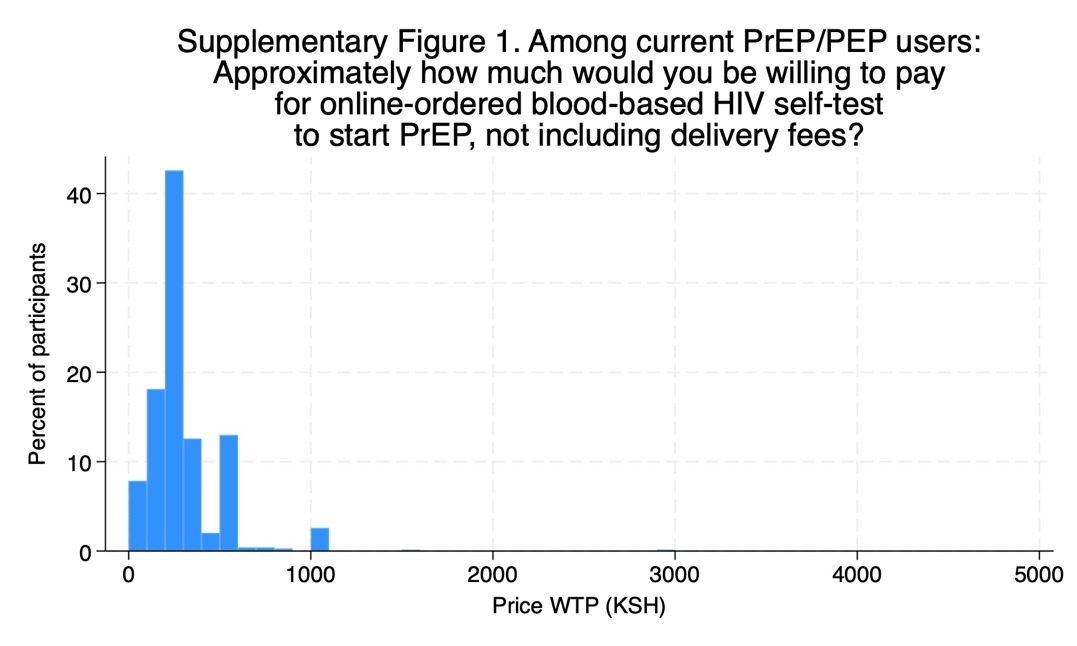

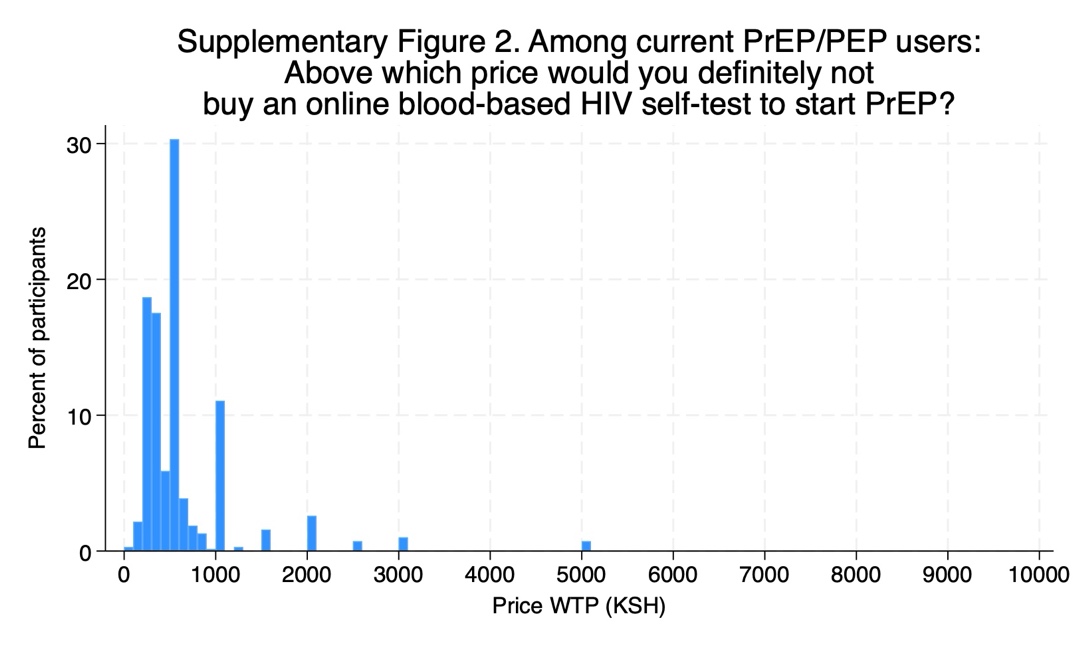


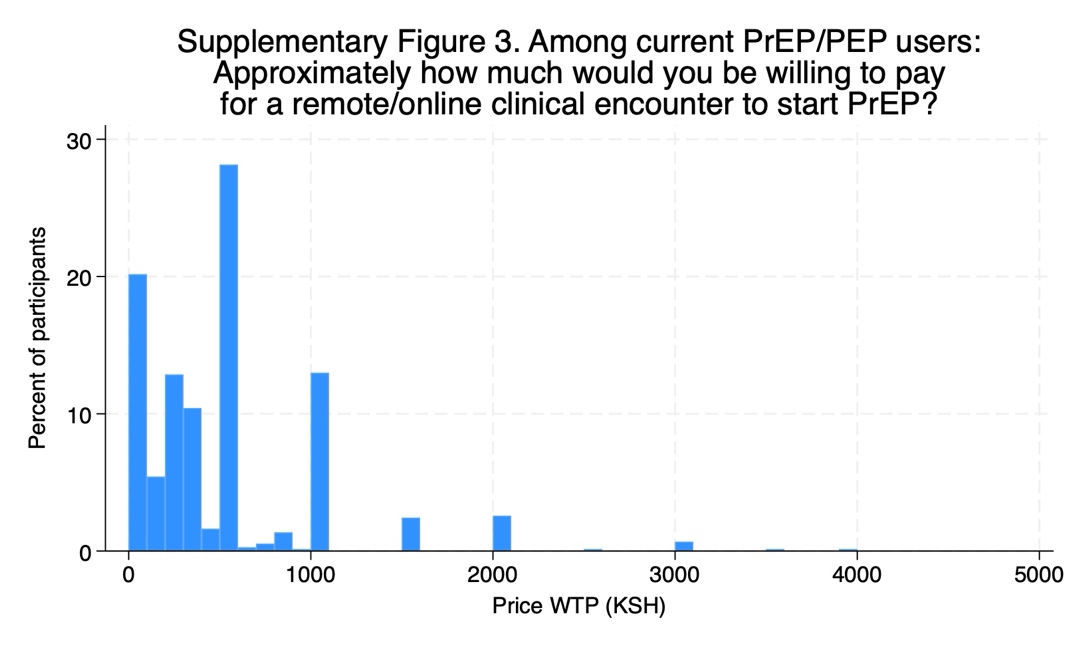

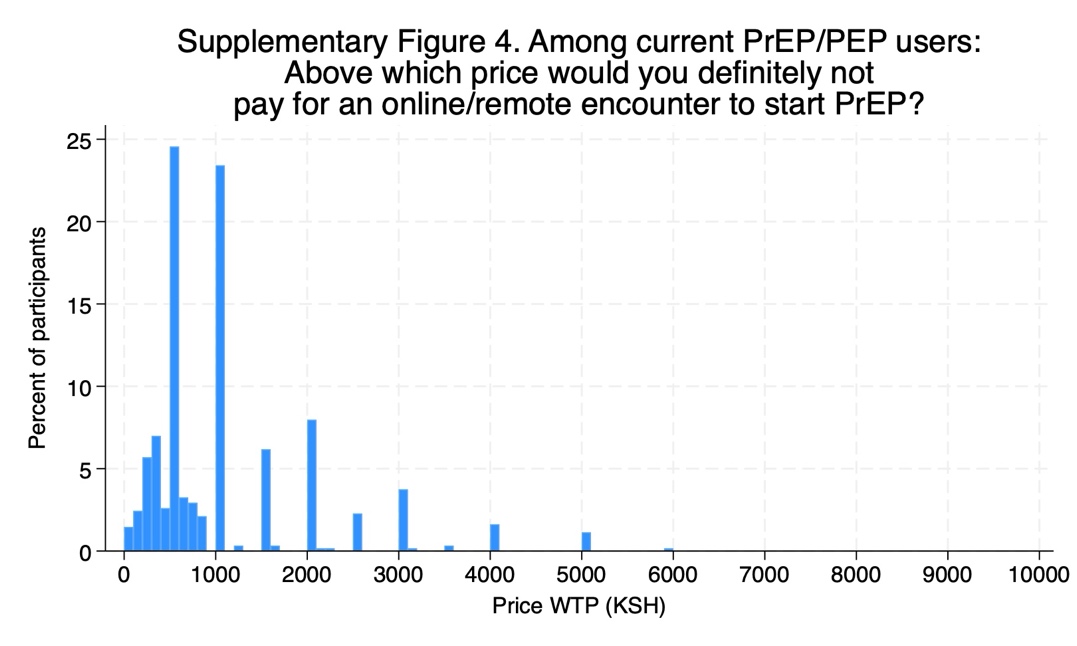


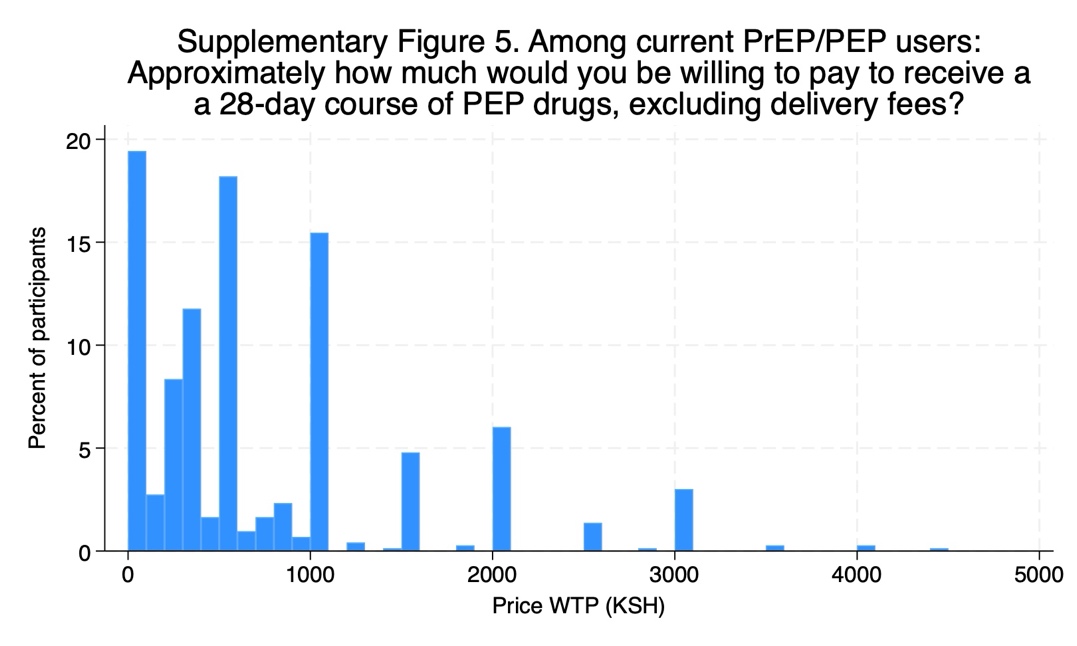

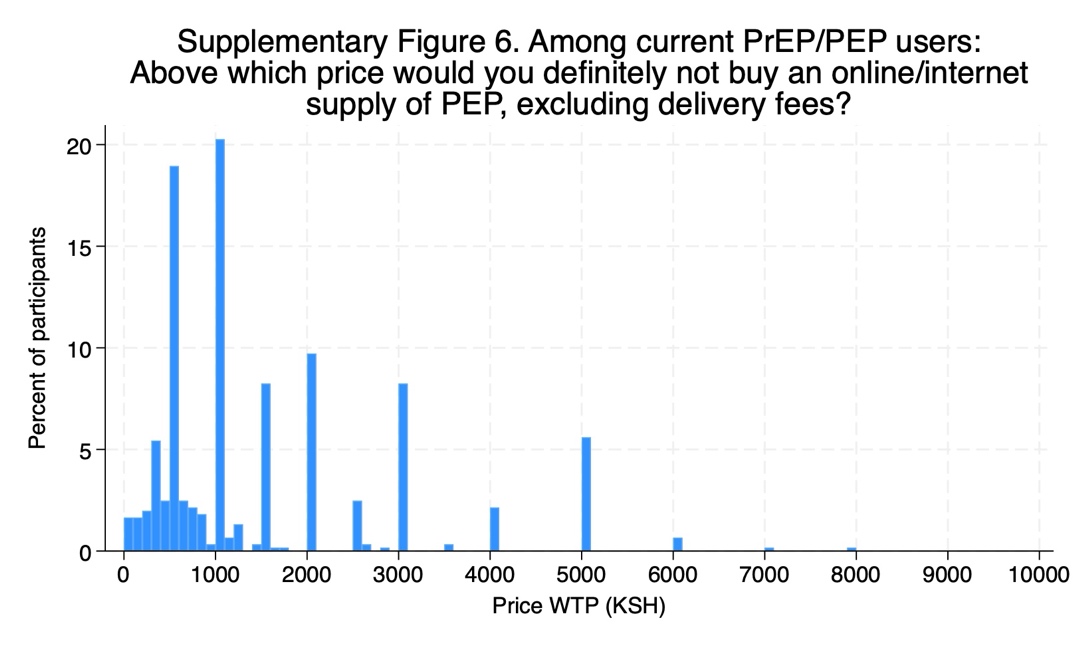


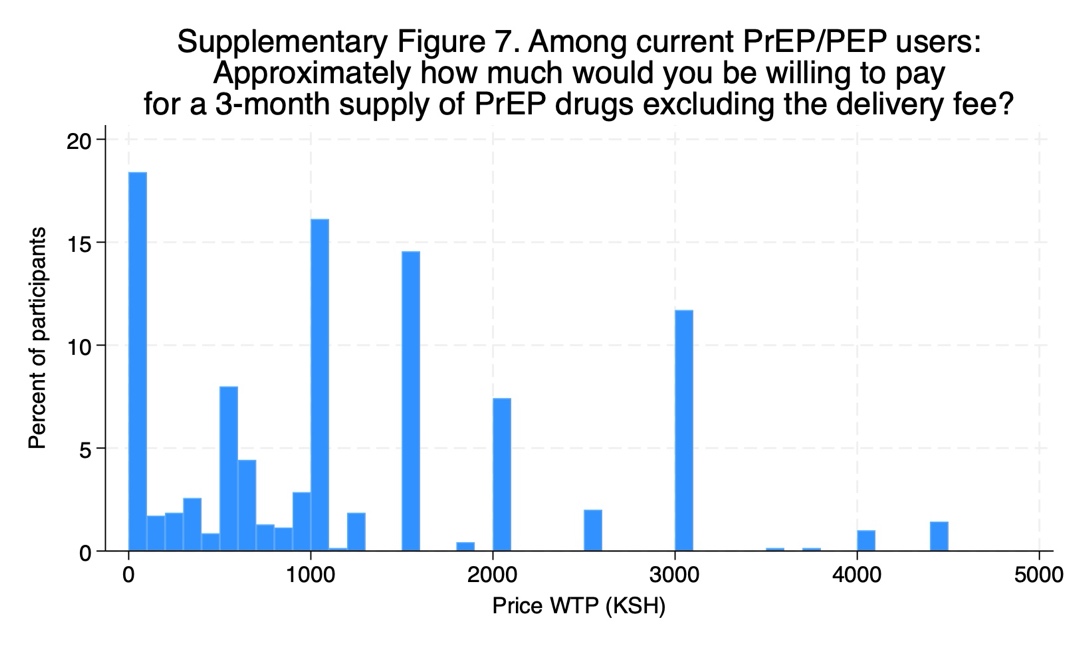

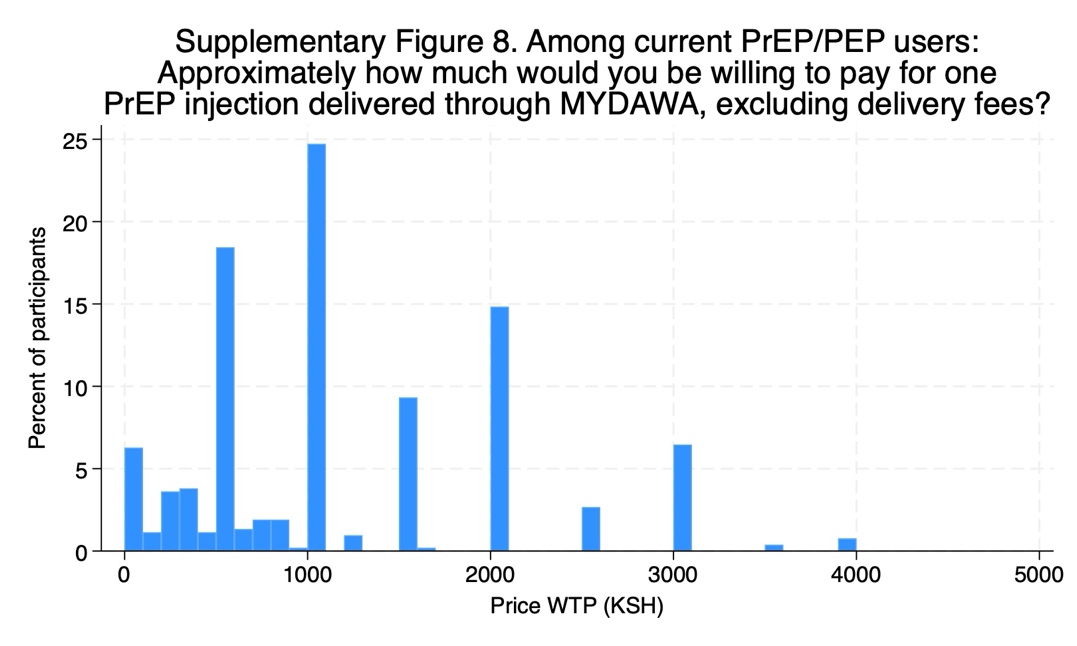


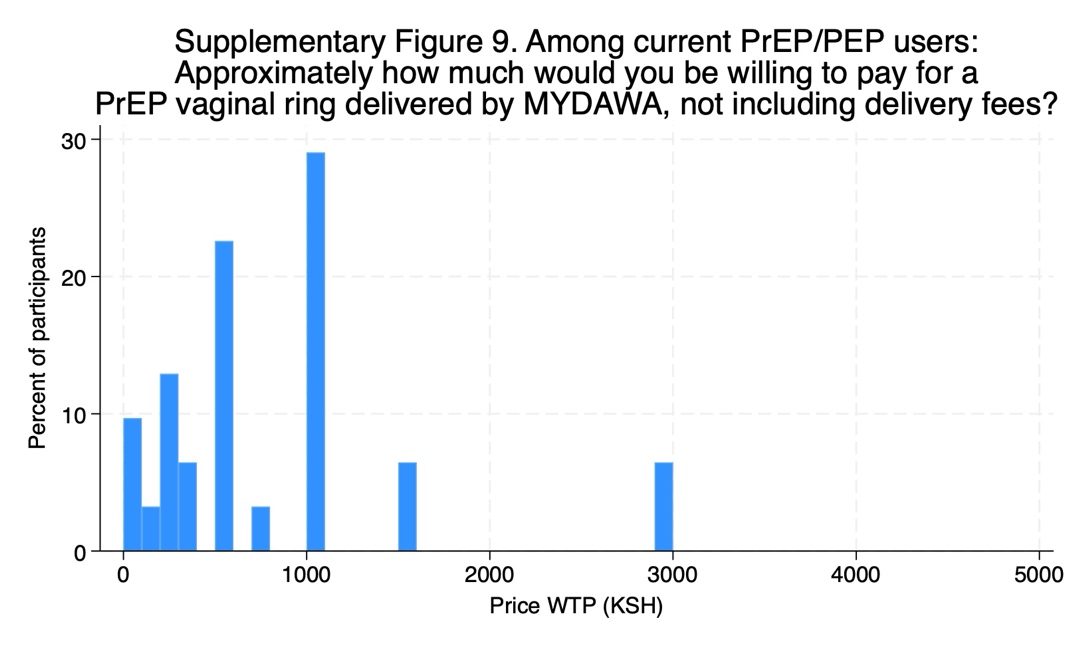


Supplementary Figures 1-9: Summary of current PrEP/PEP users’ responses to WTP questions. The amount in Kenyan shillings current PrEP/PEP users are willing to pay for blood-based HIV tests (Fig. 1), remote clinical encounter (Fig. 3), a 28-day course of PEP (Fig. 5), 3-month supply of PrEP drugs (Fig. 7), PrEP injection (Fig. 8), and PrEP vaginal ring (Fig. 9). The amount in Kenyan shillings above which current PrEP/PEP users would not purchase blood-based HIV tests (Fig. 2), remote clinical encounter (Fig. 4), and 28-day course of PEP (Fig. 6).

KSH: Kenyan shillings; PEP: Post-exposure prophylaxis; PrEP: pre-exposure prophylaxis; WTP: Willingness to pay

| **Supplementary Table 7. Summary of WTP responses from DCE questionnaire (potential PrEP users), and comparison of WTP for total package of PrEP services vs. summation of component PrEP services** | | |
| --- | --- | --- |
|  | **DCE participants**  **(N=772)** | |
| **WTP Question** | **Median (IQR)** | **Mean (SD)** |
| What is the maximum price you are willing to pay for a blood-based HIV self-test? | 200 (100-300) | 242.62 (182.66) |
| What is the maximum price you are willing to pay for an oral HIV self-test? | 200 (100-300) | 231.72 (160.79) |
| What is the maximum price you are willing to pay for remote/online clinical consultation to obtain a prescription for PrEP based on your HIV self-test results (one-time cost)? | 300 (150-500) | 362.09 (480.06) |
| What is the maximum price you are willing to pay for a one-month supply of PrEP medicines? | 300 (200-500) | 480.83 (550.89) |
| What is the maximum price you are willing to pay for delivery of PrEP medicines to a setting of your choice (one-time courier fee)? | 200 (100-200) | 192.85 (120.88) |
| What is the maximum price you are willing to pay in total for the package of services associated online PrEP delivery (this includes courier delivery of an HIV self-test, a remote clinical consultation, and courier-delivered PrEP)? | 1000 (800-1975) | 1388.41 (995.74) |
|  | | |
| Summation of component PrEP services: blood-based HIV self-test, remote clinical consultation, one-month supply of PrEP pills, courier fee | 1100 (700-1600) | 1278.39 (949.06)^b^ |
| Summation of component PrEP services: oral HIV self-test, remote clinical consultation, one-month supply of PrEP pills, courier fee | 1100 (700-1600) | 1267.49 (928.27)^c^ |
| ^a^ Percentages do not sum to 100 due to nonresponse  ^b^ Paired t-test for comparison of reported maximum WTP vs summation of PrEP components (with blood-based HIV test): p<0.001  ^c^ Paired t-test for comparison of reported maximum WTP vs summation of PrEP components (with oral HIV test): p<0.001 | | |


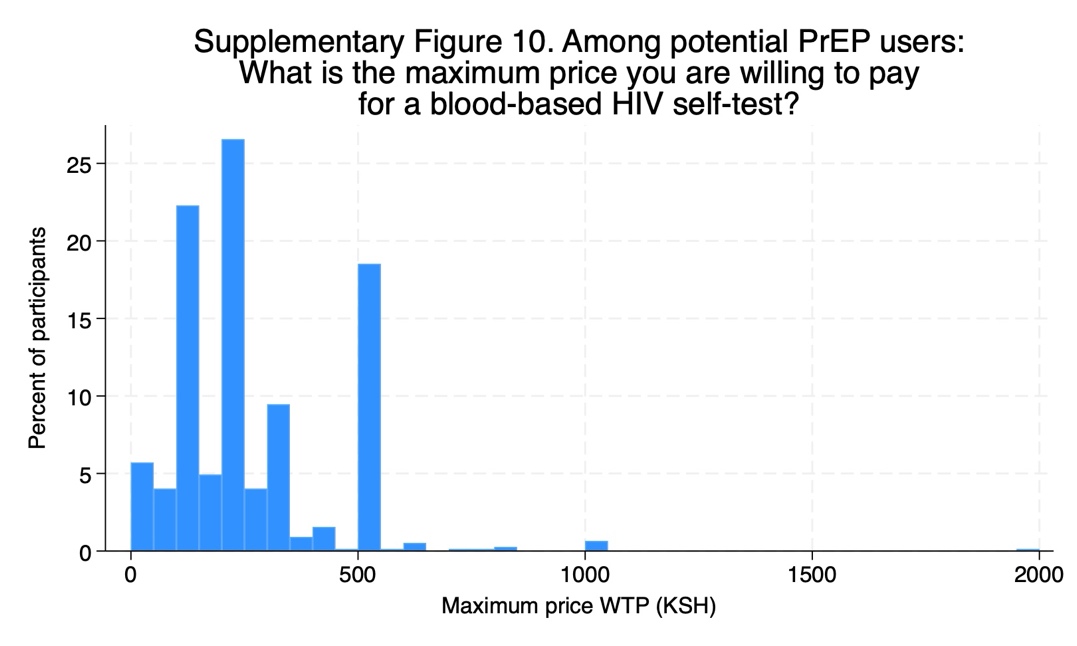

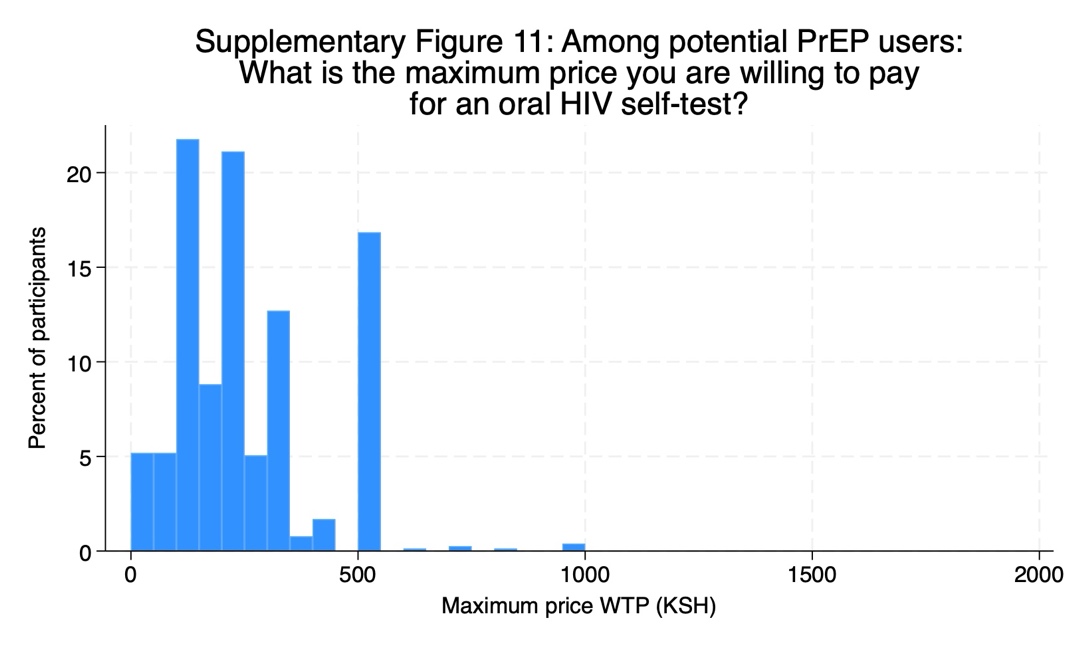


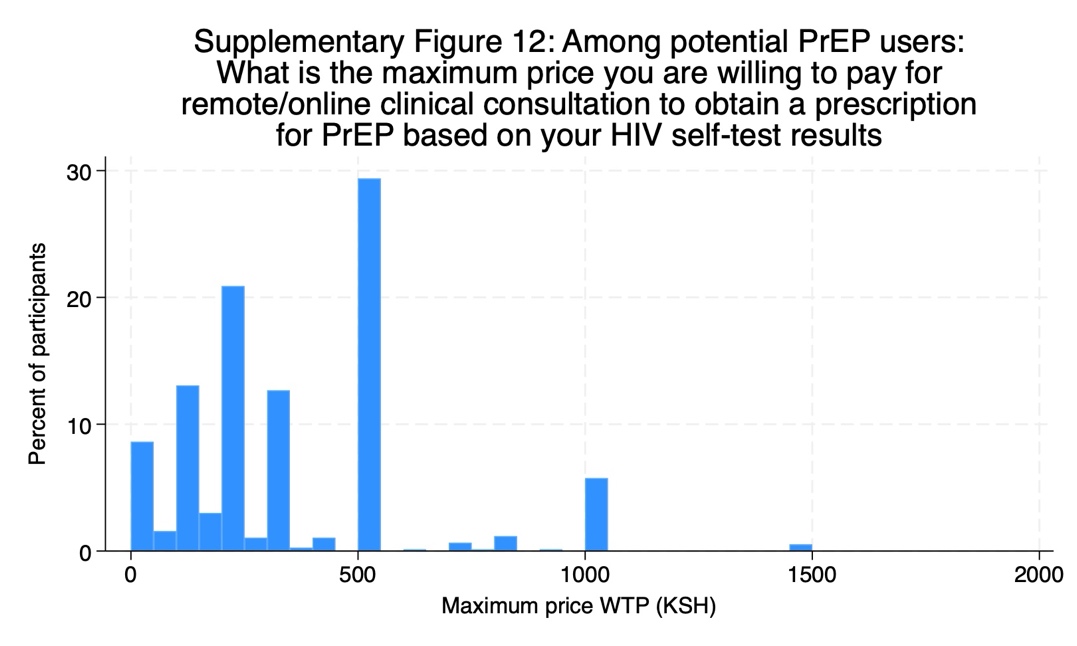

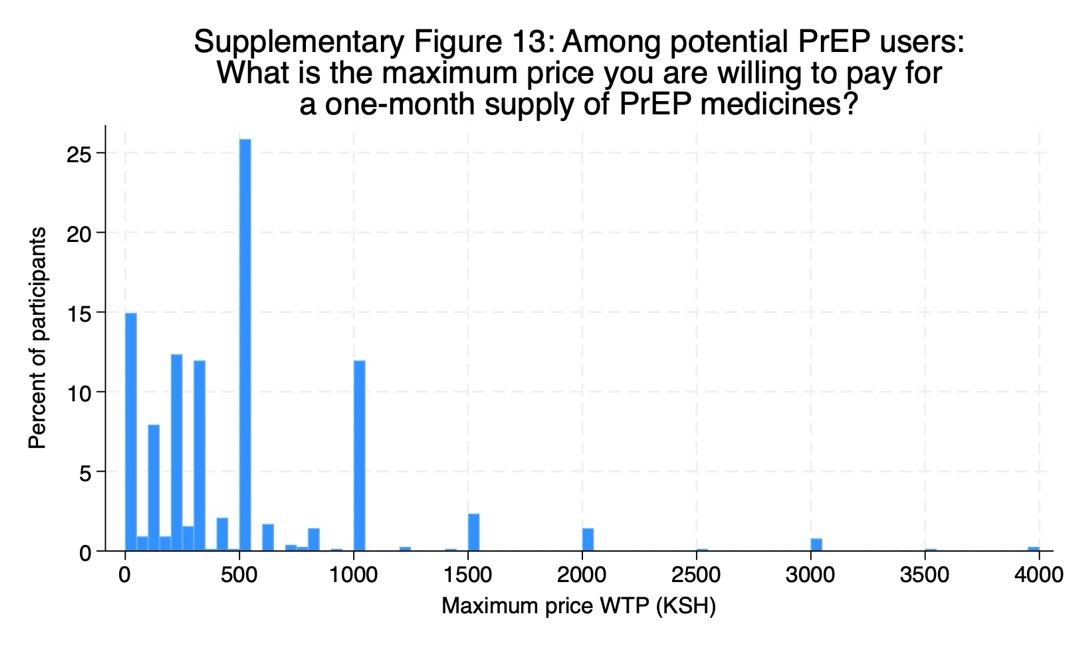


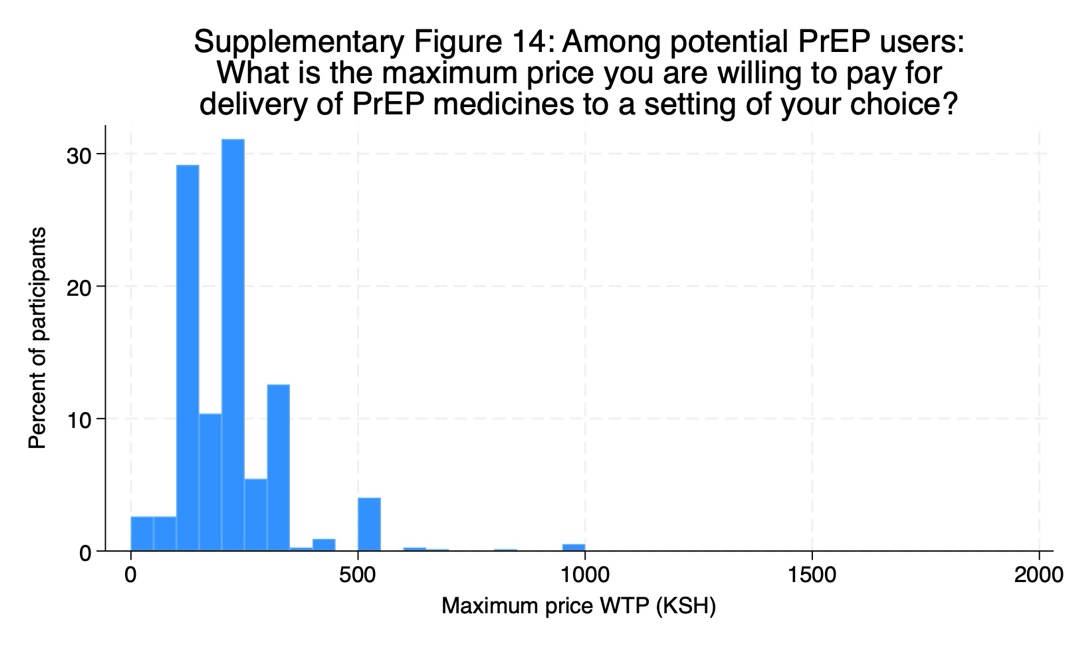

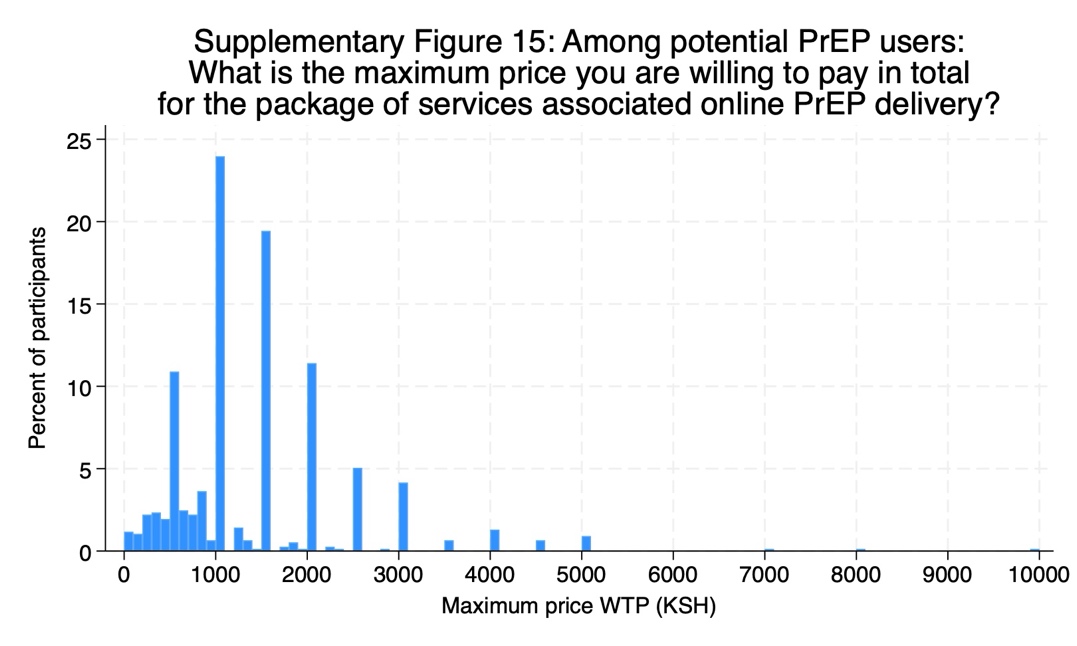


Supplementary Figures 10-15: Summary of potential PrEP users’ responses to WTP questions. The maximum amount in Kenyan shillings potential PrEP users are willing to pay for blood-based HIV tests (Fig. 10), oral HIV tests (Fig. 11), remote clinical encounter (Fig. 12), a one-month supply of PrEP drugs (Fig. 13), delivery of PrEP drugs (Fig. 5), and the total package of PrEP services, including HIV test, remote clinical encounter, one-month supply of PrEP drugs, and delivery fees (Fig. 6).

KSH: Kenyan shillings; PEP: Post-exposure prophylaxis; PrEP: pre-exposure prophylaxis; WTP: Willingness to pay

**Appendix Section 4. Bivariate models measuring associations between individual characteristics and willingness to pay**

| **Supplementary Table 8. Characteristics associated with differences in mean willingness to pay among potential online PrEP users; bivariate models** | | | | |
| --- | --- | --- | --- | --- |
|  | **WTP^a^ Mean (SD)** | **Coef.^b^** | **95% CI** | **p-value** |
| **Demographic and Socioeconomic Characteristics** |  |  |  |  |
| Age  18-24  25 or older | 1429.1 (1053.9)  1357.5 (949.3) | Ref.  0.95 | --  0.86 – 1.05 | --  0.3 |
| Sex^b^  Female  Male | 1248.3 (817.7)  1555.9 (1157.2) | Ref.  1.25 | --  1.13 – 1.38 | --  <0.001 |
| Currently Enrolled in School  No  Yes | 1321.3 (906.8)  1531.0 (1151.5) | Ref.  1.16 | --  1.04 – 1.29 | --  0.007 |
| Employment  Not employed  Part time or seasonal employment  Multiple jobs or full-time employment | 1290.8 (971.5)  1378.8 (1033.7)  1515.2 (944.6) | Ref.  1.07  1.17 | --  0.94 – 1.22  1.01 – 1.36 | --  0.3  0.03 |
| Monthly income  10,000 KSH or less  More than 10,000 KSH | 1238.2 (889.7)  1585.4 (1084.9) | Ref.  1.28 | --  1.15 – 1.42 | --  <0.001 |
| **PrEP and Online Pharmacy Engagement** |  |  |  |  |
| Ever taken PrEP  No  Yes | 1563.3 (1090.0)  1105.2 (753.8) | Ref.  0.71 | --  0.64 – 0.78 | --  <0.001 |
| Ever purchased products from an online pharmacy  No  Yes | 1306.9 (927.8)  1638.2 (1146.9) | Ref.  1.25 | --  1.12 – 1.41 | --  <0.001 |
| **Sexual Behavior** |  |  |  |  |
| Type of sexual partners, prior 3 months  Did not have sex  1 primary partner and no other partners  1 primary and 1+ casual partner(s)  1+ casual partner(s) | 1480.6 (971.9)  1508.4 (1074.1)  1293.5 (972.3)  1305.2 (905.0) | Ref.  1.02  0.87  0.88 | --  0.86 – 1.21  0.73 – 1.04  0.73 – 1.06 | --  0.8  0.1  0.2 |
| Number of sexual partners in prior 3 months  0  1  2  3  4 or more | 1460.2 (940.5)  1515.1 (1102.0)  1377.3 (1020.7)  1346.6 (838.9)  1185.7 (948.8) | Ref.  1.04  0.94  0.92  0.81 | --  0.89 – 1.21  0.80 – 1.11  0.76 – 1.12  0.68 – 0.97 | --  0.6  0.5  0.4  0.02 |
| Sex without a condom, prior 6 months  No  Yes | 1433.2 (1067.8)  1379.2 (978.2) | Ref.  0.96 | --  0.85 – 1.09 | --  0.6 |
| Exposure to HIV, prior 6 months  No  Yes  Unsure | 1405.9 (1046.1)  1375.7 (986.2)  1364.0 (873.1) | Ref.  0.98  0.97 | --  0.87 – 1.10  0.85 – 1.11 | --  0.7  0.7 |
| Diagnosis or treatment of STI, prior 6 months  No  Yes | 1402.8 (1011.1)  1305.7 (899.6) | Ref.  0.93 | --  0.80 – 1.08 | --  0.3 |
| KSH: Kenyan shillings; PrEP: pre-exposure prophylaxis; SD: standard deviation; STI: sexually transmitted infection; WTP: willingness to pay  ^a^ WTP refers to maximum willingness to pay, in Kenyan shillings, for the total package of PrEP delivery services including HIV testing, remote clinical consultation, PrEP medication, and delivery fees  ^b^ All coefficients have been exponentiated | | | | |

| **Supplementary Table 9. Characteristics associated with differences in mean willingness to pay among current online PrEP/PEP users; bivariate models** | | | | |
| --- | --- | --- | --- | --- |
|  | **Total WTP^a^ Mean (SD)** | **Coef.^b^** | **95% CI** | **p-value** |
| **Demographic and Socioeconomic Characteristics** |  |  |  |  |
| Pilot Arm  PEP Only  PrEP Only  PEP to PrEP Transition | 1275.5 (1027.4)  1373.9 (2521.7)  1083.3 (1162.0) | Ref.  1.09  0.86 | --  0.88 – 1.35  0.47 – 1.59 | --  0.4  0.6 |
| Age  18-24  25 or older | 990.1 (677.2)  1403.6 (1540.9) | Ref.  1.42 | --  1.22 – 1.65 | --  <0.001 |
| Sex  Female  Male | 1201.5 (956.3)  1315.6 (1532.4) | Ref.  1.09 | --  0.94 – 1.28 | --  0.2 |
| Currently enrolled in school  No  Yes | 1325.2 (1418.8)  1113.7 (1067.6) | Ref.  0.84 | --  0.71 – 0.99 | --  0.048 |
| Monthly income  10,000 KSH or less  More than 10,000 KSH | 878.3 (629.0)  1401.2 (1481.1) | Ref.  1.60 | --  1.35 – 1.88 | --  <0.001 |
| **PrEP, PEP, and Online Pharmacy Engagement** |  |  |  |  |
| Ever taken PrEP or PEP  No  Yes | 1276.2 (1378.9)  1232.0 (994.6) | Ref.  0.97 | --  0.76 – 1.23 | --  0.8 |
| Ever purchased products from an online pharmacy  No  Yes | 1177.0 (944.6)  1491.8 (1942.4) | Ref.  1.27 | --  1.09 – 1.48 | --  0.003 |
| **Sexual Behavior** |  |  |  |  |
| Current relationship status  Primary partner only  Casual partners only  Primary and casual partners | 1181.1 (867.6)  1446.0 (1909.5)  1283.3 (1043.7) | Ref.  0.82  0.89 | --  0.70 – 0.96  0.70 – 1.12 | --  0.01  0.3 |
| Number of sexual partners in prior 3 months  0  1  2  3  4 or more | 727.8 (796.8)  1217.1 (1636.1)  1350.1 (1095.9)  1229.1 (911.4)  1326.6 (1108.2) | Ref.  1.67  1.86  1.69  1.82 | --  0.71 – 3.97  0.78 – 4.40  0.69 – 4.12  0.74 – 4.47 | --  0.2  0.2  0.2  0.2 |
| New sexual partner in prior 3 months  No  Yes | 1269.5 (2027.3)  1282.8 (1018.6) | Ref.  1.01 | --  0.85 – 1.21 | --  0.9 |
| Sexual intercourse in prior 2 weeks  No  Yes | 1156.5 (901.3)  1414.1 (1681.7) | Ref.  1.22 | --  1.06 – 1.41 | --  0.006 |
| Condomless sexual intercourse in prior 2 weeks  No  Yes | 1209.0 (968.0)  1477.6 (2030.2) | Ref.  1.22 | --  1.04 – 1.44 | --  0.02 |
| KSH: Kenyan shillings; PrEP: pre-exposure prophylaxis; SD: standard deviation; STI: sexually transmitted infection; WTP: willingness to pay  ^a^ Total WTP was calculated for each participant by summing reported WTP for HIV testing, remote clinical consultation, and 1-month supply of PrEP medication.  ^b^ Coefficients have been exponentiated | | | | |

| **Supplementary Table 10. Bivariate models examining association between income category and willingness to pay among potential online PrEP users and current online PrEP/PEP users** | | | | | |
| --- | --- | --- | --- | --- | --- |
|  | **n (%)** | **Total WTP^a^ Mean (SD)** | **Coef.^b^** | **95% CI** | **p-value** |
| **Potential online PrEP users** |  |  |  |  |  |
| Monthly income  <10,000 KSH  10,001-30,000 KSH  30,001-50,000 KSH  >50,000 KSH | 348 (51.9)  240 (35.8)  54 (8.1)  29 (4.3) | 1238.2 (889.7)  1481.3 (943.1)  1701.9 (1306.2)  2231.0 (1471.2) | Ref.  1.20  1.37  1.80 | --  1.07 – 1.34  1.13 – 1.68  1.39 – 2.34 | --  0.002  0.002  <0.001 |
| **Current online PrEP/PEP users** |  |  |  |  |  |
| Monthly income  <10,000 KSH  10,001-30,000 KSH  30,001-50,000 KSH  >50,000 KSH | 184 (24.9)  136 (18.4)  150 (20.3)  270 (36.5) | 878.3 (629.0)  1044.8 (665.7)  1227.1 (988.1)  1677.4 (1898.7) | Ref.  1.19  1.40  1.91 | --  0.98 – 1.45  1.15 – 1.69  1.61 – 2.26 | --  0.09  0.001  <0.001 |
| KSH: Kenyan shillings; PEP: post-exposure prophylaxis; PrEP: pre-exposure prophylaxis; SD: standard deviation; WTP: willingness to pay  ^a^ Total WTP was calculated for current online PrEP/PEP users by summing reported WTP for HIV testing, remote clinical consultation, and 1-month supply of PrEP medication.  ^b^ Coefficients have been exponentiated | | | | | |
